# Quantified motility in Crohn’s disease to evaluate stricture composition using cine-MRI

**DOI:** 10.1093/bjr/tqaf120

**Published:** 2025-05-28

**Authors:** Kim J Beek, Kyra L van Rijn, Catherina S de Jonge, Floris A E de Voogd, Christianne J Buskens, Jarmila van der Bilt, Willem Bemelman, Geert D’Haens, Aart Mookhoek, E Andra Neefjes-Borst, Karin Horsthuis, Jeroen A W Tielbeek, Krisztina B Gecse, Jaap Stoker

**Affiliations:** Department of Radiology and Nuclear Medicine, Amsterdam UMC, location University of Amsterdam, Meibergdreef 9, 1105 AZ Amsterdam, The Netherlands; Amsterdam Gastroenterology Endocrinology Metabolism, Meibergdreef 9, 1105 AZ Amsterdam, The Netherlands; Department of Radiology and Nuclear Medicine, Amsterdam UMC, location University of Amsterdam, Meibergdreef 9, 1105 AZ Amsterdam, The Netherlands; Amsterdam Gastroenterology Endocrinology Metabolism, Meibergdreef 9, 1105 AZ Amsterdam, The Netherlands; Department of Radiology and Nuclear Medicine, Amsterdam UMC, location University of Amsterdam, Meibergdreef 9, 1105 AZ Amsterdam, The Netherlands; Amsterdam Gastroenterology Endocrinology Metabolism, Meibergdreef 9, 1105 AZ Amsterdam, The Netherlands; Amsterdam Gastroenterology Endocrinology Metabolism, Meibergdreef 9, 1105 AZ Amsterdam, The Netherlands; Department of Gastroenterology and Hepatology, Amsterdam UMC, location University of Amsterdam, Meibergdreef 9, 1105 AZ Amsterdam, The Netherlands; Amsterdam Gastroenterology Endocrinology Metabolism, Meibergdreef 9, 1105 AZ Amsterdam, The Netherlands; Department of Surgery, Amsterdam UMC, location University of Amsterdam, Meibergdreef 9, 1105 AZ Amsterdam, The Netherlands; Amsterdam Gastroenterology Endocrinology Metabolism, Meibergdreef 9, 1105 AZ Amsterdam, The Netherlands; Department of Surgery, Amsterdam UMC, location University of Amsterdam, Meibergdreef 9, 1105 AZ Amsterdam, The Netherlands; Department of Surgery, Flevoziekenhuis, Hospitaalweg 1, 1315 RA Almere, The Netherlands; Department of Surgery, Amsterdam UMC, location University of Amsterdam, Meibergdreef 9, 1105 AZ Amsterdam, The Netherlands; Amsterdam Gastroenterology Endocrinology Metabolism, Meibergdreef 9, 1105 AZ Amsterdam, The Netherlands; Department of Gastroenterology and Hepatology, Amsterdam UMC, location University of Amsterdam, Meibergdreef 9, 1105 AZ Amsterdam, The Netherlands; Institute of Tissue Medicine and Pathology, University Bern, Murtenstrasse 31, 3008Bern, Switzerland; Amsterdam Gastroenterology Endocrinology Metabolism, Meibergdreef 9, 1105 AZ Amsterdam, The Netherlands; Department of Pathology, Amsterdam UMC, location University of Amsterdam, Meibergdreef 9, 1105 AZ Amsterdam, The Netherlands; Amsterdam Gastroenterology Endocrinology Metabolism, Meibergdreef 9, 1105 AZ Amsterdam, The Netherlands; Department of Radiology and Nuclear Medicine, Amsterdam UMC, location Vrije Universiteit, De Boelelaan 1117, 1081 HVAmsterdam, The Netherlands; Department of Radiology and Nuclear Medicine, Spaarne Gasthuis, Boerhaavelaan 22, 2035 RCHaarlem, The Netherlands; Amsterdam Gastroenterology Endocrinology Metabolism, Meibergdreef 9, 1105 AZ Amsterdam, The Netherlands; Department of Gastroenterology and Hepatology, Amsterdam UMC, location University of Amsterdam, Meibergdreef 9, 1105 AZ Amsterdam, The Netherlands; Department of Radiology and Nuclear Medicine, Amsterdam UMC, location University of Amsterdam, Meibergdreef 9, 1105 AZ Amsterdam, The Netherlands; Amsterdam Gastroenterology Endocrinology Metabolism, Meibergdreef 9, 1105 AZ Amsterdam, The Netherlands

**Keywords:** biomarkers, magnetic resonance imaging, pathology, motility, Crohn’s disease, surgery

## Abstract

**Objectives:**

The histopathological composition of a stricture impacts clinical treatment choice. Therefore, imaging biomarkers that can distinguish a predominantly inflammatory from a chronic (i.e., non-inflammatory) stricture are highly relevant. The aim of our study was to determine whether it is possible to distinguish inflammatory (i.e., inflammatory and mixed) from chronic (i.e., non-inflammatory) strictures using quantified motility measured on cine-MRI in Crohn’s disease (CD) patients.

**Methods:**

In this prospective cross-sectional study, consecutive CD patients scheduled for small bowel resection underwent 2D cine-MRI prior to surgery. The motility of small bowel strictures and pre-stricture dilatations was quantified using a validated post-processing method (GIQuant). The resection specimens were scored by two pathologists as either: predominantly inflammatory, mixed, or predominantly chronic (i.e., non-inflammatory). For the analysis, strictures were stratified into inflammatory strictures (i.e., predominantly inflammatory and mixed) and chronic (i.e., non-inflammatory) strictures.

**Results:**

Twenty-eight patients were included with 30 strictures and 15 pre-stricture dilatations. Pre-stricture dilatation motility was higher for chronic (i.e., non-inflammatory) compared to inflammatory (i.e., inflammatory and mixed) strictures (289.5 AU [188.0-362.9] vs. 113.1 AU [83.6-142.4], *P* = .004). The area under the curve (AUC) for chronic (i.e., non-inflammatory) stricture detection was 0.93 (95% CI, 0.78-1.0, *P* = .01). Within strictures, no difference was found between motility in different histopathology categories (*P* = .6).

**Conclusion:**

Motility in the pre-stricture dilatations of chronic (i.e., non-inflammatory) strictures was higher compared to inflammatory (i.e., inflammatory and mixed) strictures. No difference in motility was observed in stricture motility among stricture subtypes. Our findings suggest that quantified motility—measured with cine-MRI—of pre-stricture dilatations could possibly distinguish chronic (i.e., non-inflammatory) strictures from inflammatory (i.e., inflammatory and mixed) strictures.

**Advances in knowledge:**

Motility measured with cine-MRI could provide an imaging biomarker for the distinction between chronic (i.e., non-inflammatory) and inflammatory (i.e., inflammatory and mixed) strictures in CD.

## Introduction

Crohn’s disease (CD) is a chronic inflammatory bowel disease characterized by episodes of relapse and remission. This pattern repeatedly activates the tissue repair mechanism that leads to an excess in the extracellular matrix with increase of mesenchymal cells, including myofibroblasts, fibroblasts, fat cells and neural hyperplasia, and smooth muscle cells.^1^ This exaggerated response leads to bowel wall thickening causing luminal narrowing, eventually resulting in intestinal strictures and dysmotility.^2^ At initial presentation, only 11% of CD patients present with an intestinal stricture; however, half develop an intestinal stricture during their disease course.^3^

Non-invasive identification of chronic (i.e., non-inflammatory) strictures (predominantly consisting of fibrosis and muscularisation, fat and nerve content rather than inflammation^4^) is crucial for clinical decision-making. For inflammatory and mixed (more or less equal inflammatory/non-inflammatory composition) strictures anti-inflammatory therapy is a treatment option, while in chronic (i.e., non-inflammatory) strictures surgical therapy is indicated.^1^^,^^5^^,^^6^

In all CD strictures inflammation, muscular hypertrophy, and fibrosis are present but to a different degree and it is challenging to determine the predominant histopathological composition with MR enterography (MRE) parameters.^7^^,^^8^ Therefore, quantitative MRI have been studied for fibrosis detection.^9–11^ The benefit of quantitative MRI techniques is that they are less subjective compared to MRE measurements. Cine-MRI is a non-invasive quantitative MRI technique where multiple MRI images are captured at the same location. This results in a time-series of multiple frames and enables quantification of bowel motility using post-processing techniques.^12^

Quantified motility measured with cine-MRI has shown a negative correlation with endoscopic and histopathological inflammatory activity—based on endoscopically retrieved biopsies—in CD.^13^ Furthermore, quantified motility was lower in small bowel strictures compared to healthy small bowel segments in patients with CD.^2^ However, stricture and pre-stricture dilatation motility has not yet been compared with histopathological stricture composition. We hypothesized that (1) stricture motility would be lower in chronic (i.e., non-inflammatory) strictures compared to inflammatory (i.e., inflammatory and mixed) strictures due to more tissue damage in chronic (i.e., non-inflammatory) strictures and (2) higher motility in the pre-stricture dilatation of chronic (i.e., non-inflammatory) strictures compared to inflammatory (i.e., inflammatory and mixed) strictures due to higher resistance of chronic (i.e., non-inflammatory) strictures.

The aim of the study was to determine whether it is possible to distinguish chronic (i.e., non-inflammatory) from inflammatory (i.e., inflammatory and mixed) strictures using quantified motility measured on cine-MRI in stricturing CD.

## Methods

### Study design

This prospective cross-sectional study (Dutch trial register NL9105: https://trialsearch.who.int/Trial2.aspx?TrialID=NL-OMON54948) was performed at a tertiary referral center (Amsterdam University Medical Center, location Academic Medical Center). Patients were recruited from December 2019 until March 2022. The study was conducted in agreement with the Declaration of Helsinki and approved by the local Medical Ethics Committee. The primary aim was to evaluate whether there was a difference in stricture and pre-stricture dilatation motility between histopathological chronic (i.e., non-inflammatory) and inflammatory (i.e., inflammatory and mixed [more or less equal inflammatory/non-inflammatory composition]) strictures. The secondary aim was to investigate the relationship between stricture and pre-stricture dilatation motility and stricture characteristics.

### Patients

Eligible adult CD patients (≥18 years) scheduled for surgical small bowel segment resection were included. For optimal correlation with the resection specimen, a maximum interval of 12 weeks between imaging and surgery was used, due to COVID this interval was allowed to be prolonged (1 patient 14 weeks, 1 patient 16 weeks, and 1 patient 25 weeks). Exclusion criteria were the presence of an isolated colonic stricture, pregnancy, inability to give informed consent, ongoing gastroenteritis, or contraindications for MRI.

### MRE protocol

Patients were prepared using the standard clinical MRE procedure. Patients fasted 4 hours before scanning and drank 1600 mL of 1.9%-mannitol solution (Baxter B.V. Utrecht, The Netherlands) within 1 hour before scanning. Images were acquired in a supine position using a 3 Tesla MRI (Ingenia, Philips, Best, and The Netherlands) with a 16-channel anterior and posterior coil. The protocol consisted of a coronal balanced Fast Field Echo (bFFE) sequence followed by an axial T2-weighted Half-Fourier Acquisition Single-shot Turbo Spin Echo without fat suppression and an axial T2-weighted Turbo Spin Echo with fat suppression. During a 20-second expiration breath-hold a coronal dynamic single slice 2D bFFE scan was acquired targeted at the stricture and the pre-stricture dilatation. These sequences were followed by a coronal T1-weighted pre-contrast and a coronal and axial post-contrast sequence (all with fat suppression), the latter after intravenous administration of 20 mg anti-peristaltic medication (scopolamine butylbromide, Buscopan; AS KALCEKS, Riga, Latvia) and 0.1 mmol/kg contrast agent (Dotarem, Gadoteric acid, Guerbet, Roissy, France).

### Stricture definitions

A stricture was defined as bowel wall thickening >3 mm and ≥50% luminal reduction compared to a normal distended bowel loop.^14^ This relatively broad definition was chosen to include a population with as many inflammatory strictures as possible. The number of pre-stricture dilatations on MRE and/or cine-MRI was documented.

### Motility measurements

The motility sequences were analysed with GIQuant (Motilent, London, UK), software using a previously validated displacement mapping technique for assessing bowel motility.^15^ First, the motility scans were checked for the presence of breathing artifacts by [CSJ] (expert in MRI motility measurements, 10 year experience) and [KJB] (clinical researcher, 3 year experience in luminal CD). Images containing breathing were removed or the entire scan was excluded when removal resulted in too little images for adequate motility measurement.^16^ Strictures and pre-stricture dilatations (immediate pre-stricture bowel with a cut-off value for luminal diameter >3 cm)^14^ were delineated on the reference image by [KJB] and supervised by [JS] (senior abdominal radiologist, 30 year of experience in luminal CD) and by [CSJ]. Motility was quantified within these regions of interest (ROI) producing a single, numerical motility score (Arbitrary Units = AU).^15^ Segments were excluded in case the segment was influenced by motility of adjacent bowel loops.

### MRE parameters

MRE scans were independently assessed by 2 abdominal radiologists with special interest in luminal CD (JT and KH with 14 and 21 years of experience, respectively). The radiologists were blinded for all clinical information and for the cine-MRI. Parameters were assessed per small bowel segment; if >5 cm unaffected small bowel was present between affected segments the adjacent segment was assigned as a second segment. Assessed parameters and definitions were summarized in Table 1. The formula used for calculation of percentage of luminal narrowing was [(1 − (diameter of most narrowed lumen/diameter of lumen of normal bowel loop)) × 100]. Discrepancies between the readers were resolved in a consensus meeting. Discrepancies in bowel wall thickness and percentage of luminal narrowing were only discussed if this resulted in a difference in cut-off value (e.g., >3 mm and <3 mm). The percentage of luminal narrowing was used as a continuous outcome for comparison with motility, and as a dichotomous outcome for interobserver agreement due to the cut-off value of >50% luminal narrowing for defining a stricture.^14^ For (percentage of) bowel wall thickness, (percentage of) luminal narrowing and stricture length (continuous) the mean between two readers was used as definitive value. Disease activity of the segmented pre-stricture dilatations was scored by [JS] using the simplified Magnetic Resonance Index of Activity (sMARIA) score.^17^ sMARIA ≥1 was defined as active disease.^17^ All researchers involved in imaging analysis were blinded to histopathology findings.

**Table 1.** MRI parameters scored on conventional static MRI sequences.

Parameter	Categories	Definition
Stricture length	0-5 cm	Total length of the stricture measured in cm.
	5-15 cm	When the disease-free distance between multiple strictures was more than 5 cm, a stricture was classified as a second stricture; otherwise, it was added up.
	15-30 cm	
	30-50 cm	
	>50 cm	
Luminal narrowing	Measured in continuous (%) for comparison with motility	Measured in % compared to an adjacent appropriately distended normal bowel loop^14^ measured on a fat-suppressed T1-weighted post-contrast sequence.
	Measured dichotomous for interobserver agreement	A luminal reduction of at least 50% was defined as narrowing.
	≥50% luminal narrowing	
	<50% luminal narrowing	
Presence of a pre-stricture dilatation	Present	Luminal diameter >3 cm,^14^ if so the maximum diameter was measured.
	Absent	
Bowel wall thickness	Measured in mm	>3 mm^26^
		Measured as the thickest part of the bowel wall on axial fat-suppressed T1-weighted post-contrast or fat-suppressed T2-weighted sequence.
Total length of diseased small bowel	0-5 cm	Bowel wall thickness >3 mm and the presence of contrast enhancement on a fat-suppressed T1-weighted post-contrast sequence.^a^
	5-15 cm	
	15-30 cm	
	30-50 cm	
	>50 cm	
Fatty wrapping	Present	Scored as present when more than 50% of the bowel wall surface was covered with mesentery.^27^
	Absent	
Mesenteric inflammation	Normal	Increase in mesenteric signal and/or fluid rim assessed on T2-weighted fat saturated sequence.
	Increase in mesenteric signal but no fluid	
	Small fluid rim ≤ 2 mm	
	Larger fluid rim >2 mm.^28^	
Comb sign	Present	Engorged vasa recta that supply an inflamed loop evaluated on a fat-suppressed T1-weighted post contrast sequence, when not available evaluated on a bFFE sequence.
	Absent	
Presence of fistulas	Present	The presence of a simple or complex fistula.^26^
	Absent	
Presence of abscesses	Present	Encapsulated fluid collection with rim enhancement on MR enterography, with or without internal gas measured on a fat-suppressed T1-weighted post-contrast sequence.
	Absent	

If a skip lesion of more than 5 cm was present and an upstream or downstream segment had disease activity it was scored as a second affected segment.

### Histopathology

The fresh resection specimen was opened, and pins were placed to indicate the locations that matched the most narrowed small bowel on MRE, as assessed by researchers (KJB and KR, clinical researcher with 6 year experience in MRI of luminal CD). Matching was based on the distance at MRE between the most narrowed part and ileocecal valve or anastomosis in cm. After formalin fixation, tissue blocks were taken at the pin-marked areas. The tissue was processed to produce tissue sections stained with hematoxylin and eosin (H&E). Two experienced gastrointestinal pathologists, blinded to all clinical and imaging findings, with 33 years [AN] and 9 years [AM] of experience in CD, independently scored the slides as (1) predominantly inflammatory, (2) predominantly chronic (i.e., non-inflammatory) or (3) mixed phenotype. The determination of phenotype was based on the relative contribution to wall thickness of either active or chronic inflammation, fibrosis, adipose tissue, and/or muscular hypertrophy. In case of the absence of both active and chronic inflammation, presence of fibrosis, and/or adipocytes in the submucosa in combination with muscular hypertrophy of the *muscularis mucosae* and *muscularis propria* was scored as chronic. In the presence of significant chronic and/or active inflammation (significant neutrophilic or lymphocytic/plasmocytic infiltrate), the extent of chronic changes present influenced whether a slide was scored as mixed or inflammatory. In case of discrepancy in phenotype scored between the two pathologists, a consensus meeting was held. For data analyses the consensus score was used.

To accurately determine the definitive histopathology category of the stricture (given the intrinsic heterogeneity of small bowel CD strictures), a primary histopathological category was scored in a variable number of tissue blocks within the stricture (number of tissue blocks dependent on stricture length). In the case of multiple primary histopathological categories in the stricture, the stricture was determined to be mixed. Inflammatory and mixed strictures were taken together as inflammatory and compared with chronic (i.e., noninflammatory) strictures.

### Statistical analysis

All motility scores were presented as median [IQR]. For comparisons between motility with histopathology a Kruskal-Wallis test and a Mann-Whitney *U*-test were used. The area under the ROC curve (AUC) was used to determine the accuracy. Correlations were analysed by mean of a Spearman’s rank correlation test. Interobserver agreement for continuous variables was performed by means of an Intraclass Correlation Coefficient (ICC) with a two-way random model for absolute agreement. For categorical variables a weighted kappa statistic was used with quadratic weights. If weighted kappa statistic was not possible to perform, due to zero variance in ratings for one or two observers, results were presented in percentage of agreement. For histopathological interobserver agreement, the primary histopathological category was used. For the comparison of motility with clinical parameters per patients’ analysis was performed. This implies that the segment closest to the ileocecal valve was chosen because this segment was affected in most patients. A sensitivity analysis for the primary outcome, accounting for potential confounding from through-plane motion caused by motility was included in Figure S1. No power calculation was performed upfront because of the exploratory nature of this study. *P*-values lower than .05 were considered as statistically significant.

## Results

### Study population

In total 32 patients with stricturing small bowel CD fulfilled the inclusion criteria. Four patients were excluded: (1) no histopathological tissue block was taken at the most narrowed segment on cine-MRI, (2) uncorrectable breathing artefact present, (3) reliable matching between MRI and histopathological resection specimen was not feasible and (4) resection specimen only consisted out of colon tissue. One stricture and one pre-stricture dilatation were excluded because of the influence of the motility of an adjacent bowel loop. Baseline characteristics are presented in Table 2.

**Table 2.** Baseline characteristics of patients with small bowel stricturing Crohn’s disease.

Characteristics in median [IQR], mean (±SD) or *n* (%)	Patients with stricturing CD (*n* = 28)
Sex (*n*, % female)	15 (54%)
Age (years)	35.0 [23.5-55.5]
**Smoking status**	
Never smoked	17 (61%)
Previous smoker	8 (29%)
Current smoker	3 (11%)
BMI in kg/m^2^	24.2 [20.4-27.6]
Harvey Bradshaw Index	7.0 [3.5-9.8]
**Crohn’s Disease Obstructive Score**	4.0 [0.0-5.0]
Nausea and vomiting	13 (46%)
Dietary restrictions	13 (46%)
Hospitalization due to obstruction	1 (4%)
Disease duration (yrs)	7.0 [3.3-14.3]
**Anti-inflammatory medication**	
None	8 (29%)
Corticosteroids monotherapy or add-on	1 (3%)/5 (18%)
Thiopurines	5 (18%)
Add-on methotrexate	2 (7%)
Biologicals	14 (50%)
**Types of surgery**	
Ileocecal resection	18 (64%)
Ileocecal reresection	9 (32%)
Ileocecal resection and small bowel segment resection	1 (4%)
Time between MRI and surgery (days)	20.5 [4.5-55.0]
**Surgical history**	9 (32%)
Ileocecal resection	9 (32%)
Ileocecal reresection	1 (4%)
Stricturoplasty	1 (4%)
Subtotal colectomy	1 (4%)
History of endoscopic dilatation	4 (14%)
**Disease location**	
L1, ileal	12 (43%)
L2, colonic	0
L3, ileocolonic	16 (57%)
+ perianal disease	6 (21%)
**Disease behaviour**	
B1, Non-stricturing/non-penetrating	2 (7%)
B2, Stricturing	22 (79%)
B3, Penetrating	4 (14%)
Faecal calprotectin (µg/L) (*n =* 16)	806.5 [233.3.0-1606.8]
CRP (mg/L) (*n* = 23)	6.2 [2.3-13.1]
Patients who underwent endoscopy last 6 months, *n* (%)	15 (54%)
Stricture on endoscopy, *n* (%)	12 (43%)
Possibility to intubate stricture, *n* (%)	4 (14%)

### Stricture characteristics

Thirty strictures were analysed in 28 patients. In 19 of these 30 strictures (63%) a pre-stricture dilatation was present. Of these 19 segments, 13 (68%) had a pre-stricture dilatation on both cine-MRI and MRE and 2 (11%) only on cine-MRI; the other 4 (21%) were only assessable on MRE (2 were not present in the FOV on cine-MRI and 2 were not captured at the center of the pre-stricture dilatation and/or were influenced by the motility of an adjacent bowel loop). In 6 of the 15 segmented pre-stricture dilatations disease activity was present, of which 5 were in the inflammatory (i.e., inflammatory and mixed) group on a total of 8 pre-stricture dilatations (5/8) and 1 in the chronic (i.e., non-inflammatory) group on a total of 7 (1/7).

No difference between diameter of pre-stricture dilatations of chronic (i.e., non-inflammatory) and inflammatory (i.e., inflammatory and mixed) strictures was found (30.9 mm [30.5-46.3] vs. 36.4 mm [33.2-48.0], *P* = .3). Table 3 displays stricture characteristics of measured MRE.

**Table 3.** Stricture characteristics on conventional MRI.

Characteristics in median [IQR] or *n* (%)	Strictures (*n* = 30)
**Stricture length**	
*0-5 cm*	11 (37%)
*5-15 cm*	16 (53%)
*15-30 cm*	2 (7%)
*30-50 cm*	1 (3%)
*>50 cm*	-
Luminal diameter stricture	2.0 mm [1.4-2.5]
Percentage of luminal narrowing	90.3% [84.8-92.8]
Presence of a pre-stricture dilatation	17 (57%)
Median diameter of pre-stricture dilatation	33.0 mm [32.0-37.3]
Bowel wall thickness	7.5 mm [7.0-9.1]
**Total length of diseased small bowel**	
*0-5 cm*	3 (10%)
*5-15 cm*	16 (53%)
*15-30 cm*	4 (13%)
*30-50 cm*	6 (20%)
*>50 cm*	1 (3%)
Fatty wrapping	28 (93%)
**Mesenteric inflammation**	
*Normal*	12 (40%)
*Increase in mesenteric signal but no fluid*	9 (30%)
*Small fluid rim*	3 (10%)
*Large fluid rim*	6 (20%)
Comb sign	25 (83%)
**Presence of fistulas**	2 (7%)
*Simple fistula*	1 (3%)
*Complex fistula*	1 (3%)
Presence of abscesses	1 (3%)

### Histopathology

Most strictures were mixed (*n* = 15, 50%) followed by chronic (i.e., non-inflammatory; *n* = 11, 37%) and inflammatory (*n* = 4, 13%) (Table S1). Table S1 shows the distribution and number of tissue blocks per stricture with the primary and definitive pathological category.

### Motility measurements

The strictures (*n* = 30) had a median ROI size of 9.3 cm^2^ [6.0-16.4] and a median motility score of 70.2 AU [61.6-92.8]. The pre-stricture dilatations (*n* = 15) had a median ROI size of 13.3 cm^2^ [10.8-23.8] and a median motility score of 160.0 AU [108.1-298.0].

### Comparison of motility measurements with histopathological stricture type

**Figure 1.**
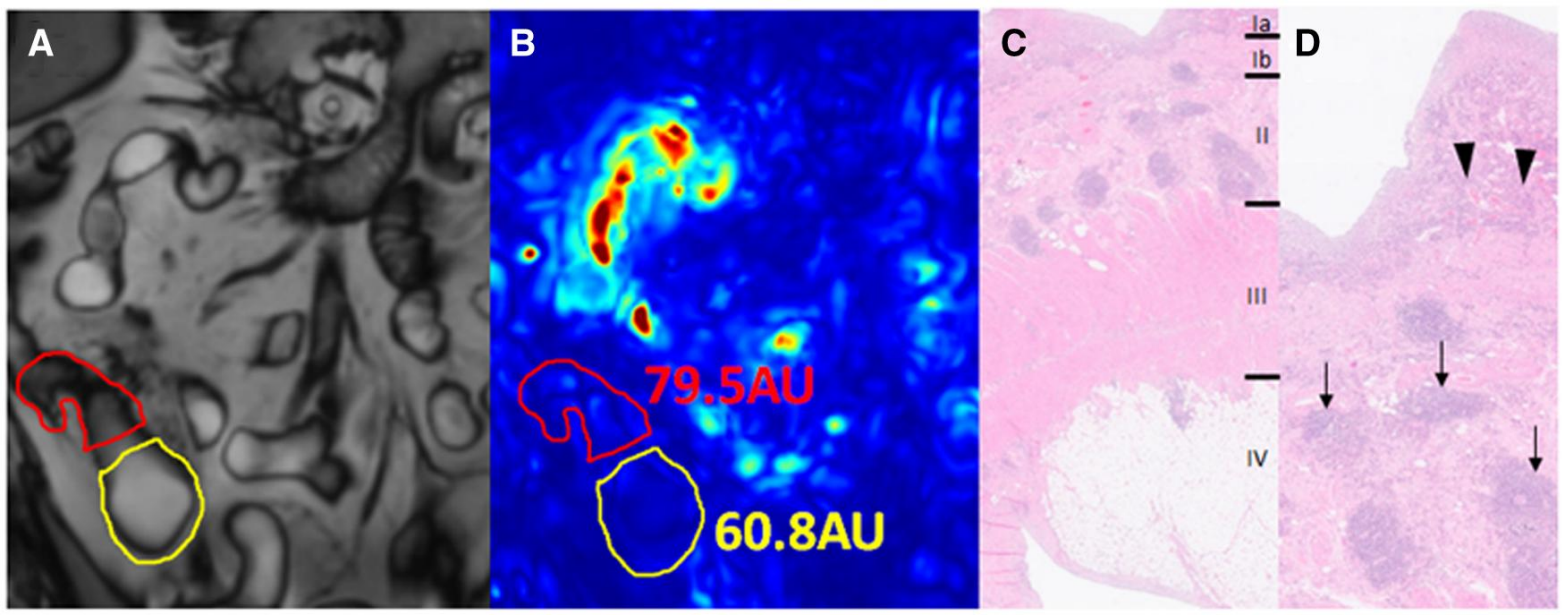
Examples of a coronal cine MRI (A) of pre-stricture dilatation (yellow segmentation) and stricture (red and blue segmentation, respectively), with corresponding quantified motility scores (in AU) (B) and histopathology (C and D) in an inflammatory (Fig. 1A-D) and a chronic (i.e., non-inflammatory) stricture (Fig. 2A-D). (A) Example of an inflammatory stricture (in red) with corresponding pre-stricture dilatation (in yellow) on the reference image, (B) motility map with motility score of inflammatory stricture (79.5AU) and pre-stricture dilatation (60.8AU) and (C) histopathology (H&E stain) shows the different bowel wall layers: Ia mucosa, Ib muscularis mucosae, II submucosa III muscularis propria, IV mesenteric fatty tissue, and (D) detailed image with mucosal inflammation (arrowheads) and submucosal aggregates of lymphocytes (arrows).

**Figure 2.**
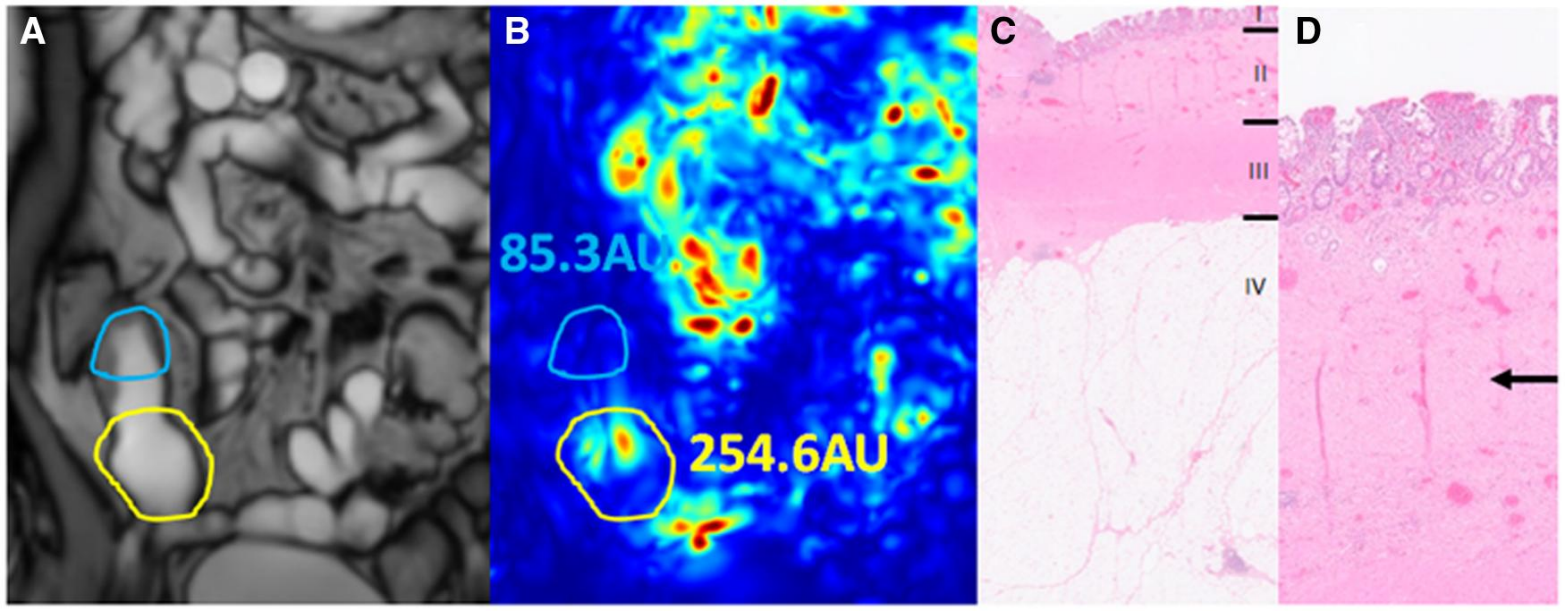
Examples of a coronal cine MRI (A) of pre-stricture dilatation (yellow segmentation) and stricture (red and blue segmentation, respectively), with corresponding quantified motility scores (in AU) (B) and histopathology (C and D) in an inflammatory (Fig. 1A-D) and a chronic (i.e., non-inflammatory) stricture (Fig. 2A-D). (A) Example of a chronic (i.e., non-inflammatory) stricture (in blue) with corresponding pre-stricture dilatation (in yellow) on the reference image, (B) motility map with motility score of chronic (i.e., non-inflammatory) stricture (85.3AU) and pre-stricture dilatation (254.6AU) and (C) Histopathology (H&E stain) shows the different bowel layers: I mucosa, II submucosa (the muscularis mucosae is indistinguishable from the submucosa), III muscularis propria, IV mesenteric fatty tissue (d) detailed image of submucosal fibrosis (arrow).

**Figure 3.**
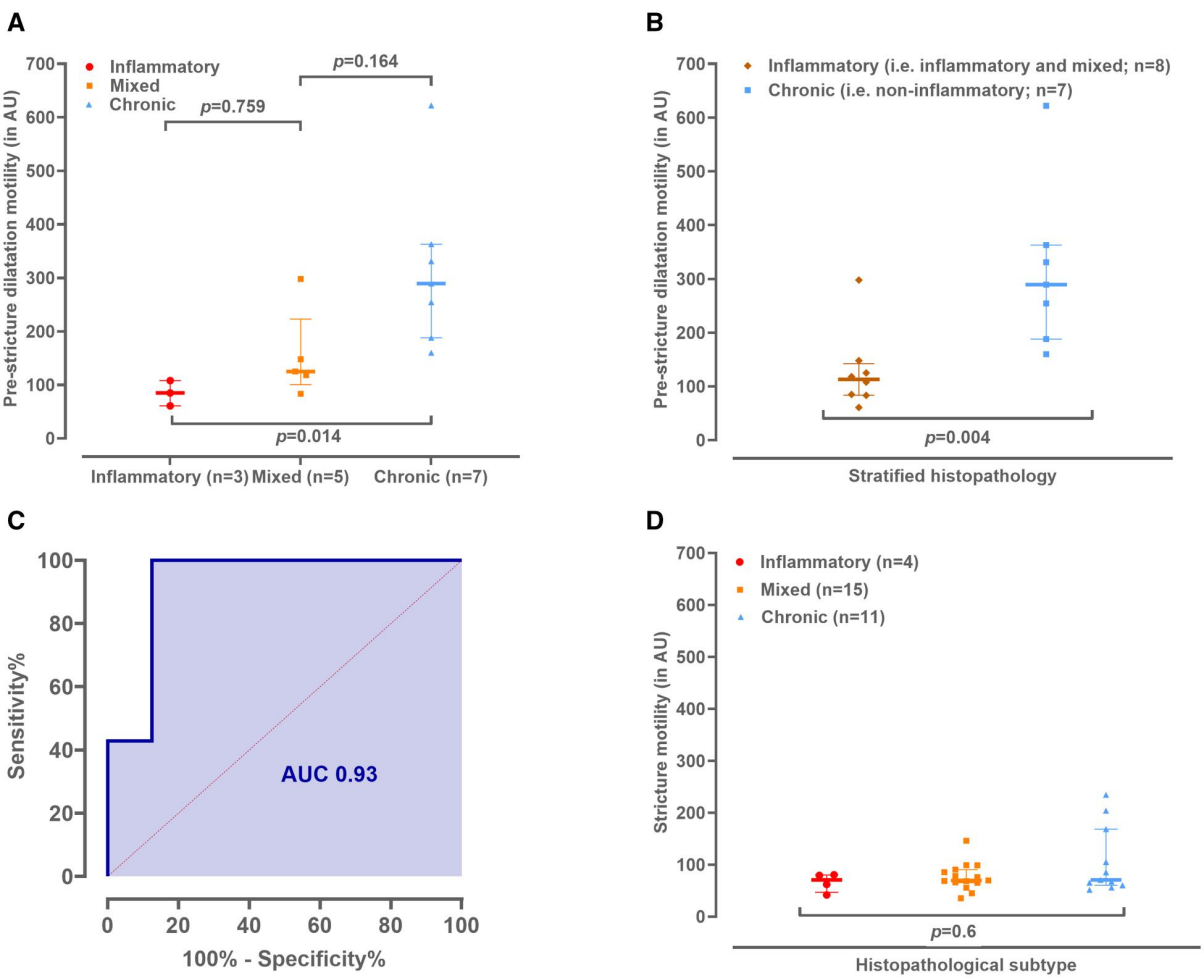
(A) Pre-stricture dilatation motility in AU per histopathological category, (B) pre-stricture dilatation motility in AU stratified for inflammatory (i.e., inflammatory and mixed) versus chronic (i.e., non-inflammatory), (C) area under the curve (AUC) for detection of chronic (i.e., non-inflammatory) strictures with pre-stricture dilatation motility. (D) Stricture motility in AU per histopathological category.

In pre-stricture dilatations of inflammatory strictures (*n* = 3) motility was 85.0 AU [60.8-108.1], of mixed strictures (*n* = 5) this was 125.1 AU [100.6-223.1] and of chronic (i.e., non-inflammatory) strictures (*n* = 7) this was 289.5 AU [188.0-362.9]. Figures 1 and 2 demonstrate an example of an inflammatory stricture and a chronic (i.e., non-inflammatory) stricture with corresponding pre-stricture dilatation and histopathology. A significantly higher pre-stricture dilatation motility was found for chronic (i.e., non-inflammatory) strictures compared to inflammatory strictures (*P* = .014, Figure 3A). After stratification, the motility index for pre-stricture dilatation of chronic (i.e., non-inflammatory) strictures was significantly higher compared to the inflammatory (i.e., inflammatory and mixed) strictures (289.5 AU [188.0-362.9] vs. 113.1 AU [83.6-142.4], *P* = .004) (Figure 3B). Pre-stricture dilatation motility for distinguishing chronic (i.e., non-inflammatory) from inflammatory (i.e., inflammatory and mixed) yielded an AUC of 0.93 (95% CI 0.78-1.0, *P* = .01, Figure 3C).

Stricture motility scores did not differ betwen inflammatory, mixed, or chronic (i.e., non-inflammatory) strictures (*P* = .6, Figure 3D).

After excluding pre-stricture dilatations that presented with through-plane motion on MRI due to motility, sensitivity analysis showed no differences (Figure S1).

### Comparison of motility measurements with stricture characteristics

**Figure 4.**
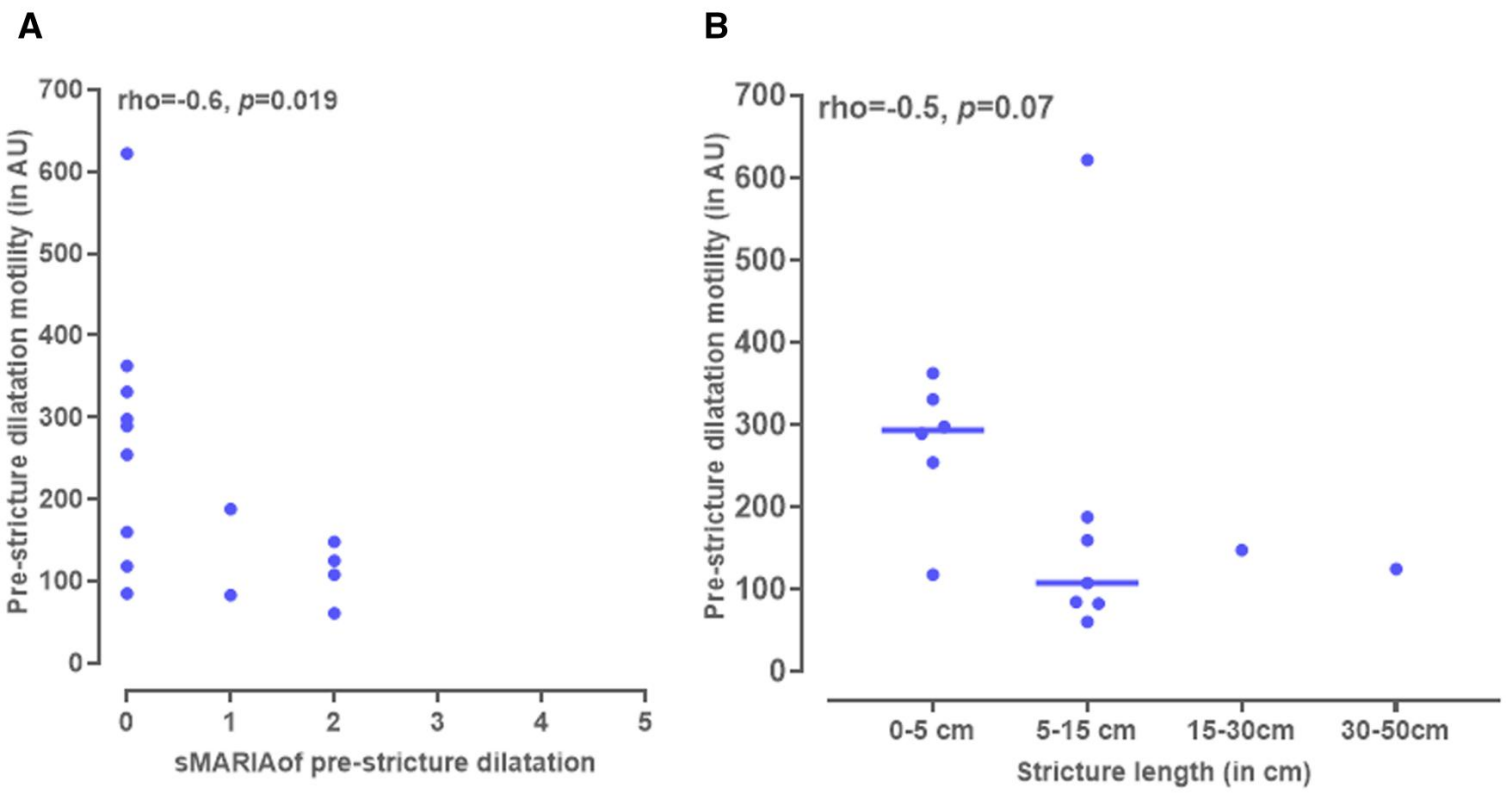
Pre-stricture dilatation motility correlated with pre-stricture dilatation inflammatory activity measured with sMARIA score (A) and stricture length (B).

A negative correlation was found between pre-stricture dilatation motility and pre-stricture dilatation sMARIA (Figure 4A). A trend between pre-stricture dilatation motility and stricture length was found (Figure 4B). For other stricture characteristics, no correlations were found with pre-stricture dilatation motility or stricture motility (Table S2A and B).

### Interobserver agreement conventional MRE parameters

Interobserver agreement for MRE (Table 4) ranged from fair to almost perfect.^18^

**Table 4.** Interobserver agreement for conventional MRI parameters.

MRI feature	ICC, κ [95 CI%] or percentage of agreement	Interpretation^a^
Stricture length	0.21 [−0.02 to 0.45]	Fair
Presence of pre-stricture dilatation	0.36 [0.11-0.61]	Fair
Diameter of pre-stricture dilatation	0.21 [−0.12 to 0.58]	Fair
Bowel wall thickness	0.48 [0.15-0.72]	Moderate
Fatty wrapping	0.53 [0.08-0.97]	Moderate
Mesenteric inflammation	0.61 [0.34-0.88]	Substantial
Comb sign	0.63 [0.30-0.96]	Substantial
Presence of fistulas	1.0 [1.0-1.0]	Almost perfect
Total disease length of diseased small bowel	0.83 [0.74-0.91]	Almost perfect
Presence of abscesses	97%	
≥50% percentage of luminal narrowing	100%	

a Cohen’s κ interpretation according to Landis and Koch^18^: <0.00 refers to poor agreement, 0.00-0.20 to slight agreement, 0.21-0.40 to fair agreement, 0.41-60 to moderate agreement, 0.61-80 to substantial agreement, and 0.81-1.00 to almost perfect agreement.

### Interobserver agreement histopathology

Interobserver agreement for histopathology (Table S1) was substantial.^18^

### Comparison of motility with clinical parameters

For both stricture as pre-stricture dilatation motility no correlations were found with nausea and vomiting, Harvey Bradshaw Index, disease duration, Crohn’s disease obstructive score, faecal calprotectin, and CRP.

## Discussion

This study investigated the ability of quantified motility to distinguish chronic (i.e., non-inflammatory) from inflammatory (i.e., inflammatory and mixed) strictures. Between strictures, no difference in motility was found. Interestingly, we found higher motility in pre-stricture dilatations of chronic (i.e., non-inflammatory) compared to inflammatory (i.e., inflammatory and mixed) strictures. This could mean that the motility in a pre-stricture dilatation could provide information on the composition of strictures. In this exploratory study, we showed a high accuracy of motility scores in a pre-stricture dilatation for detection of chronic (i.e., non-inflammatory) strictures. To our knowledge, this is the first study to use cine-MRI motility measurements to distinguish chronic (i.e., non-inflammatory) and inflammatory (i.e., inflammatory and mixed) strictures in CD patients undergoing surgery.

An explanation for higher pre-stricture dilatation motility of chronic (i.e., non-inflammatory) strictures could be that chronicity in strictures, which primarily presents as fibrosis and muscular hypertrophy,^19^ leads to higher stricture stiffness. This hypothesis is supported by two studies showing a higher shear wave speed in ultrasound elastography for patients who underwent surgical resection or had predominantly fibrotic strictures, compared wtih patients who did not undergo surgical resection or had predominately inflammatory strictures.^20^^,^^21^ As a response to higher tissue stiffness, the proximal bowel could possibly compensate with higher motility in the pre-stricture dilatation to be able to push the luminal content through the “stiffer” stricture.

Another possible explanation could be that disease activity extends in the pre-stricture dilatation of inflammatory (i.e., inflammatory and mixed) strictures, leading to a lower motility score of the pre-stricture dilatation. Only six of the 15 segmented pre-stricture dilatations showed disease activity (sMARIA) of which five in the inflammatory (i.e., inflammatory and mixed) group. Disease activity may be a confounding factor resulting in a lower motility score. Also, stricture length may influence pre-stricture dilatation motility. Furthermore, it is important to mention that pre-stricture dilatation motility could depend not only on histopathology but also on other factors such as stricture characteristics (e.g., degree of luminal narrowing).

We did not find a difference in stricture motility among different histopathological stricture subtypes. However, we know from literature that small bowel motility in CD is decreased in both strictures and in bowel inflammation.^2^^,^^13^ This indicates that differences in stricture motility within different histopathological subtypes are challenging to detect, also considering the limited number of inflammatory strictures in this study. A possible explanation for the lack of difference in stricture motility among different histopathological subtypes could be a decrease of as well interstitial cells of Cajal^22^ as telocytes^23^ (involved in gastrointestinal motility^24^). Two studies in CD patients showed a decrease in interstitial cells of Cajal^22^ and telocytes^23^ in small bowel (obstructive) resection specimen both compared with unaffected samples (of an external population). Also, the extent of damage to the interstitial cells of Cajal was not associated with disease duration.^22^ However, it is important to realise that disease duration of a specific small bowel segment is hard to establish. Another explanation could be that if there are motility differences between histopathological stricture subtypes (edema vs. fibrosis/muscularisation), these differences may be too small to be detected by the GIQuant motility quantification.

Our reported motility in strictures was lower compared to a previous study.^2^ This might be due to a selection bias towards patients who were scheduled for a surgical segment resection. Furthermore, Menys et al^2^ did not include histopathology as a reference; therefore, the proportion of chronic (i.e., non-inflammatory) strictures is unknown. In contrast to those results, we were not able to confirm that the more dilated the small bowel was, the lower the motility score was.^2^

One strength of this study is the inclusion of a histopathological reference that was scored by two independent pathologists who were blinded to clinical and MRI results. Also, we were able to include patients with inflammatory small bowel strictures that had the possibility to be treated by a relatively early ileocecal resection based on shared decision making.^25^ Nevertheless, the number of mixed and chronic (i.e., non-inflammatory) strictures included in our study was considerably higher than the number of solely inflammatory strictures.

The main limitation of our study is the relatively small sample size, which not only hampers drawing firm conclusions but also hinders the interpretation and correction for confounding factors. Second, our study is limited by the lack of a validated histopathological scoring index for small bowel strictures in CD. Therefore, we developed a scoring system. A third limitation is the use of 2D cine-MRI, which hampered the inclusion of four pre-stricture dilatations. Furthermore, motility scores could be influenced by through-plane motion in small segmentations. However, we demonstrated with a sensitivity analysis that our results were not impacted by the inclusion of segments with through-plane motion caused by motility.

In conclusion, our findings suggest that quantified motility of pre-stricture dilatations could possibly distinguish chronic (i.e., non-inflammatory) from inflammatory (i.e., inflammatory and mixed) strictures. The quantitative nature of motility scores would allow for defining a cut-off value. Confirmation of our results in a larger external cohort, also looking at confounding factors, is pivotal to draw solid conclusions for clinical guidance. This could aid gastroenterologists and surgeons in their decision-making whether to treat a stricture with anti-inflammatory treatment or surgical segment resection or, in future, anti-fibrotic treatment.

## Supplementary Material

tqaf120_Supplementary_Data

## Data Availability

The data underlying this article will be shared on reasonable request to the corresponding author.
