# Supplementary material for: Quantified motility in Crohn’s disease to evaluate stricture composition using cine-MRI
Source: Br J Radiol. 2025 May 28;98(1172):1245–54. doi: 10.1093/bjr/tqaf120 (PMC12341669; doi:10.1093/bjr/tqaf120)
Supplement: tqaf120_Supplementary_Data [file tqaf120_supplementary_data.zip › tqaf120_Supplementary_Data/Suppl_material_clean16052025.docx]

**Supplementary tables and figures**

*Supplementary table 1.* *Distribution and number of tissue blocks with primary and definitive pathological categories*

| Number of tissue blocks per stricture | Number of strictures | Primary pathological tissue present in strictures | Definitive pathological categories |
| --- | --- | --- | --- |
| 1 | 21 (70%) | Inflammatory (n=4)  Mixed (n=9)  Chronic (n=8) | Inflammatory (n=4)  Mixed (n=9)  Chronic (n=8) |
| 2 | 7 (23%) | Chronic (n=3)  Mixed (n=1)  Inflammatory and mixed (n=1)  Mixed and chronic (n=2) | Chronic (n=3)  Mixed (n=4) |
| 3 | 2 (7%) | Inflammatory and mixed (n=1)  Inflammatory, mixed and chronic (n=1) | Mixed (n=2) |
| Total |  |  |  |
| 41 tissue blocks  Cohen’s kappa:  0.69 [95%CI 0.53-0.84) | 30 strictures | Inflammatory (n=4)  Mixed (n=10)  Chronic (n=11)  Inflammatory and mixed (n=2)  Mixed and chronic (n=2)  Inflammatory, mixed and chronic (n=1) | Inflammatory (n=4)  Mixed (n=15)  Chronic (n=11) |

*Supplementary figure 1. Sensitivity analysis for a. Pre-stricture dilatation motility (in AU) per histopathological subtype b. Pre-stricture dilatation motility for inflammatory (i.e. inflammatory and mixed) versus chronic (i.e. non-inflammatory) strictures*

*Supplementary table 2a. Correlations between pre-stricture dilatation motility and stricture characteristics*

| Stricture characteristics | Correlation coefficient (rho) | *p*-value |
| --- | --- | --- |
| Length/luminal diameter ratio (stricture) | -0.306 | 0.268 |
| Stricture length (categorial) | -0.486 | 0.066 |
| Stricture length (continuous) | -0.324 | 0.239 |
| Luminal diameter stricture (in mm) | 0.220 | 0.430 |
| Percentage luminal narrowing stricture | -0.270 | 0.331 |
| Bowel wall thickness stricture (in mm) | -0.168 | 0.550 |
| Percentage of bowel wall thickening stricture | -0.168 | 0.550 |
| Diameter pre-stricture dilatation (on cine-MR) | 0.014 | 0.960 |
| Disease duration in years | -0.197 | 0.481 |
| Pre-stricture segment Disease activity (sMARIA) | -0.598 | 0.019 |

*Supplementary table 2b. Correlations between stricture motility and stricture characteristics*

| Stricture characteristics | Correlation coefficient (rho) | *p*-value |
| --- | --- | --- |
| Length/luminal diameter ratio (stricture) | -0.077 | 0.323 |
| Stricture length (categorial) | -0.287 | 0.125 |
| Stricture length (continuous) | -0.048 | 0.799 |
| Luminal diameter stricture (in mm) | 0.175 | 0.356 |
| Percentage luminal narrowing stricture | -0.086 | 0.650 |
| Bowel wall thickness stricture (in mm) | -0.187 | 0.323 |
| Percentage of bowel wall thickening stricture | -0.187 | 0.323 |
| Diameter pre-stricture dilatation (on cine-MR) | -0.182 | 0.516 |
| Disease duration in years | 0.081 | 0.672 |
| Pre-stricture segment Disease activity (sMARIA) | -0.428 | 0.111 |
