# Supplementary figures and images for: Quantified motility in Crohn’s disease to evaluate stricture composition using cine-MRI
Source: Br J Radiol. 2025 May 28;98(1172):1245–54. doi: 10.1093/bjr/tqaf120 (PMC12341669; doi:10.1093/bjr/tqaf120)

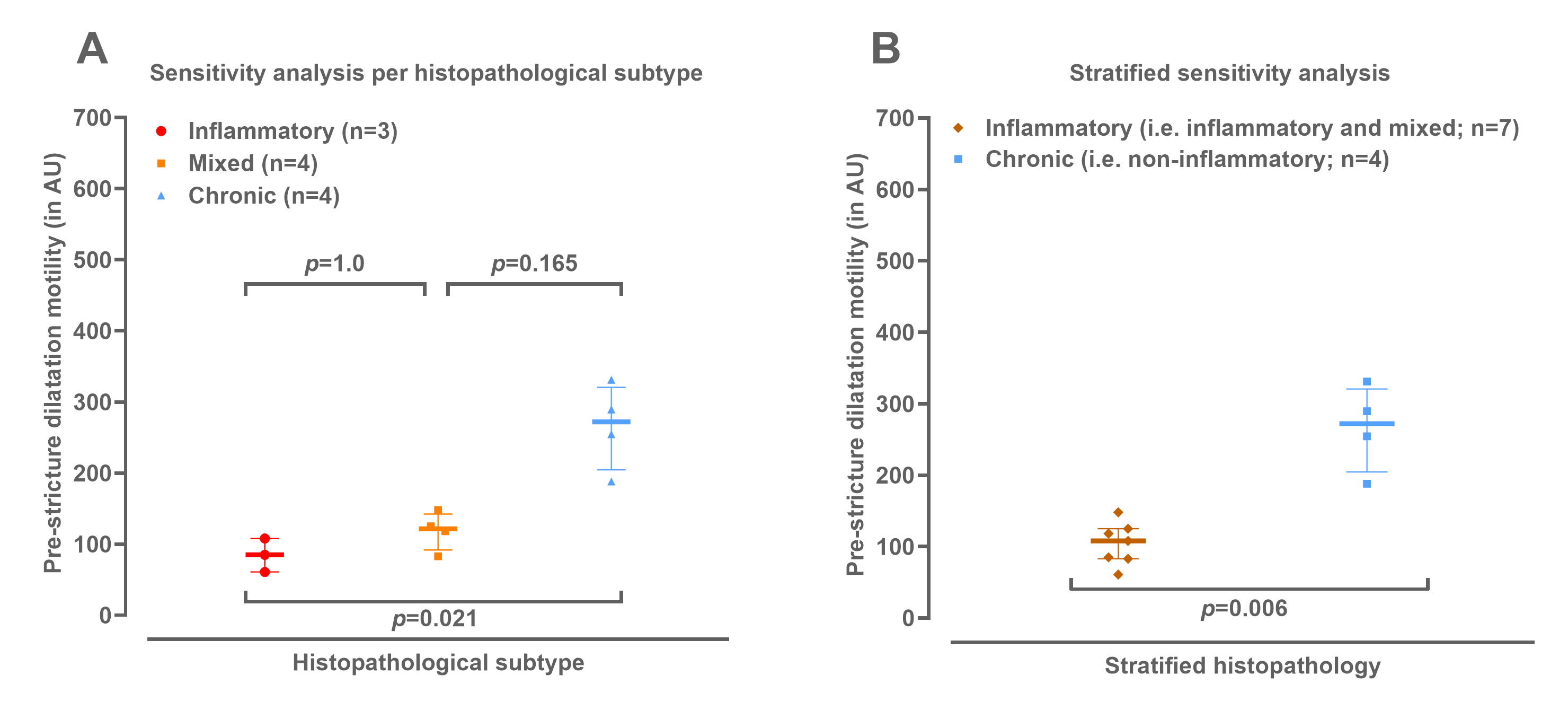

Supplement: tqaf120_Supplementary_Data [file tqaf120_supplementary_data.zip › tqaf120_Supplementary_Data/FigureS1_corr.tif]
